# Increasing Fluid Viscosity Ensures Consistent Single-Cell Encapsulation

**DOI:** 10.1021/acs.analchem.3c05243

**Published:** 2024-04-22

**Authors:** Emile Pranauskaite, Valdemaras Milkus, Justas Ritmejeris, Rapolas Zilionis, Linas Mazutis

**Affiliations:** Institute of Biotechnology, Life Sciences Center, Vilnius University, Vilnius LT 10257, Lithuania

## Abstract

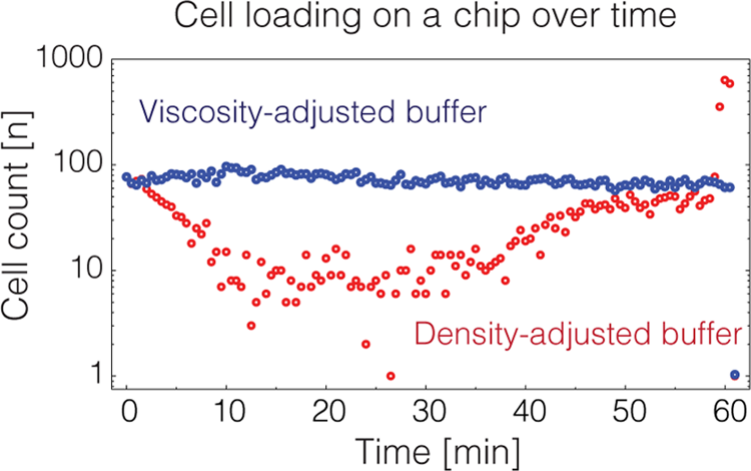

High-throughput single-cell analysis typically relies on the isolation of cells of interest in separate compartments for subsequent phenotypic or genotypic characterization. Using microfluidics, this is achieved by isolating individual cells in microdroplets or microwells. However, due to cell-to-cell variability in size, shape, and density, the cell capture efficiencies may vary significantly. This variability can negatively impact the measurements and introduce undesirable artifacts when trying to isolate and characterize heterogeneous cell populations. In this study, we show that single-cell isolation biases in microfluidics can be circumvented by increasing the viscosity of fluids in which cells are dispersed. At a viscosity of 40–50 cP (cP), the cell sedimentation is effectively reduced, resulting in a steady cell flow inside the microfluidics chip and consistent encapsulation in water-in-oil droplets over extended periods of time. This approach allows nearly all cells in a sample to be isolated with the same efficiency, irrespective of their type. Our results show that increased fluid viscosity, rather than cell-adjusted density, provides a more reliable approach to mitigate single-cell isolation biases.

Microfluidics has emerged as a pivotal technology with applications across biology, biochemistry, and biomedicine. One of the key features offered by microfluidics is the possibility of conducting high-throughput analyses on single cells or biomolecules within microreactors in the pico- to nanoliter volume range.^1^ Among diverse microfluidic systems reported to date, droplet-based single-cell analytical methods have gained a particularly broad interest and utility.^2−4^ While considerable progress has been made in the development of molecular biology workflows,^5^ there has been relatively little attention on achieving efficient and unbiased capture of cells constituting the heterogeneous populations.

In a typical scenario, microfluidics-based single-cell isolation involves a preparatory step where the cells of interest at first are dispersed in an aqueous buffer (i.e., phosphate-buffered saline, PBS supplemented with bovine serum albumin, BSA) and subsequently are loaded into a microfluidic device for further processing and analysis. However, because of density mismatch, the cells tend to sediment in the aqueous medium at different rates, creating a challenge for unbiased isolation of individual cells. Uncontrolled cell sedimentation can be particularly problematic for applications that require extended times of microfluidic operations (≥15 min), for example, single-cell nucleic acid barcoding and sequencing. One common approach to mitigate this issue is to increase the density of the fluid,^6^ yet finding the optimum that would accommodate the wide range of cell densities, shapes, and sizes is difficult to achieve. Active measures such as stirring^1,7^ add technical complexity to the experimental setup and may damage fragile cells. Thus, finding a robust approach for consistent and unbiased cell loading during microfluidic operations could have important implications for diverse single-cell screening applications.

In this work, we investigate cell loading into a microfluidics device and subsequent single-cell encapsulation in water-in-oil droplets using fluids with increased viscosity and density. We built a microfluidics setup that allows us to inject cell suspensions without leaving a dead volume and track the cell flow dynamics with high temporal resolution. We demonstrate that increasing the viscosity of the aqueous medium rather than altering its density offers a more effective means to overcome uncontrolled cell sedimentation and loss. In the viscosity-adjusted fluids, single-cell isolation becomes steady and uniform over an extended period of time. These findings were reproducible on different types of cells with different sizes, densities, and shapes. Finally, we validated the reverse transcription (RT), polymerase chain reaction (PCR), and single-cell RT-PCR assays in the presence of biopolymers, which help overcome cell sedimentation. The results of our work could benefit a wide range of biological applications that rely on uniform and unbiased single-cell isolation and analysis.

## Experimental Section

### Reagents

The biopolymer fraction in a solution is reported in % (w/v) unless stated otherwise. Dextran, 350–550 kDa (Serva Feinbiochemica); xanthan gum, 4500 kDa (Sigma-Aldrich); methylcellulose, ∼86 kDa (Sigma-Aldrich); OptiPrep (Progen); mineral oil (Sigma); cell culturing medium and supplements (Gibco): RPMI 1640 medium, IMDM medium, DMEM, 10% (v/v) fetal bovine serum (FBS), penicillin (10,000 units/mL)–streptomycin (10,000 μg/mL) solution (PS), 10× Dulbecco’s phosphate-buffered saline (Thermo Scientific), and TrypLE express enzyme (Thermo Scientific); Maxima H minus reverse transcriptase (Thermo Scientific); RiboLock RNase inhibitor (Thermo Scientific); KAPA 2× HiFi Hot Start PCR (Kapa biosystem); 10 mM dNTP mix (Thermo Scientific); 2× Maxima SYBR green/ROX qPCR Master Mix (Thermo Scientific); microfluidics consumables (Atrandi Biosciences), droplet stabilization oil (Atrandi Biosciences); and Platinum Taq (Thermo Scientific), Dream Taq (Thermo Scientific), pBR322 plasmid (Fisher Scientific), Maxima SYBR Green/ROX qPCR Master Mix (2×) (Thermo Scientific), Igepal CA-630 (Sigma-Aldrich), and Triton X-100 (Sigma-Aldrich) were used in the study.

### Cell Lines

9e10, K562, Ker-CT, and A549 cells (ATCC) were used in the study.

### Cell Culture and Preparation

K562 suspension lymphoblast cells (ATCC) and 9e10 semiadherent mouse hybridoma cells (ATCC) were cultured in RPMI 1640 and IMDM mediums, respectively, both supplemented with 10% (v/v) FBS and 1× PS. The cells were harvested at 37 °C/5% CO_2_ until confluence. Before each experiment, the semiadherent cells (9e10) were washed in 1× DPBS solution, trypsinized with TrypLE for 5 min, pelleted at 300g for 5 min, washed three times in 1× PBS, and kept on ice. Suspension K562 cells were washed three times in 1× DPBS and then kept on ice.

### Cell Loading into the Microfluidics Device

At first, the cells were resuspended in 1× PBS buffer supplemented with a corresponding amount of biopolymers to obtain the cell concentration of ∼1 mln/mL. Next, the cell suspension was withdrawn into a poly(tetrafluoroethylene) (PTFE) tubing (0.56 mm inner diameter and 1.07 mm outer diameter) and connected to a 1 mL syringe (Injekt, Braun) prefilled with mineral oil. Within a 5 min window, the syringe was mounted on a syringe pump (Harvard Apparatus) and primed, and cell infusion initiated at a flow rate of 100 μL/h. The tubing carrying cell suspension was kept straight along the gravitational axis, as indicated in Figure S1, and was directly connected to the inlet of the microfluidics chip, placed on a microscope stage. The microfluidics chip was made of PDMS elastomer bound to the 25 × 75 mm glass slide and comprised rectangular microchannels of 80 μm height.

### Droplet Generation and Single-Cell Encapsulation

Droplet generation and cell encapsulation were performed on a custom-built microfluidics platform and open-source system Onyx (Atrandi Biosciences), using a microfluidics device having a nozzle 70 μm wide and 80 μm deep. The flow rates used were 100 μL/h for aqueous solution and 300 μL/h for droplet stabilization oil. Droplet generation on-chip was recorded by using a high-speed camera Phantom V7.0. For single-cell isolation experiments, the cell suspensions comprising ∼105 cells/100 μL were loaded in 1 nL droplets using the same flow rates. The encapsulated cells were collected in a 1.5 mL tube and subsequently analyzed on a hemocytometer under the bright field microscope.

### Monitoring Cell Loading and Cell Flow Dynamics

The still images (4908 × 3264 pixels) of cells passing through the microfluidics device were recorded under a Nikon Eclipse Ti-E microscope equipped with a Nikon DS-Qi2 digital camera and 10× objective (CFI Plan Fluor 10×, N.A. 0.30, W.D. 16.0 mm). The time-lapse images were cropped and converted into .png files using the Python programming language. Next, cell numbers were extracted using the Ilastik machine-learning algorithm.^8^ The algorithm is trained to recognize cells and count them using Pixel and Object classifications. The cells that were attached to the surface of the microfluidic device or did not move over time were excluded from the cell count. The obtained numbers of recognized cells were further analyzed by using Python. The cell sedimentation (decay rate) in different biopolymer solutions was obtained by fitting the exponential function. The cell clumps and aggregates (*n* > 2) were excluded from the analysis.

### Osmolarity Measurements

The osmolarity of 1× PBS buffer having different amounts of biopolymers was measured using a Gonotec Osmomat Freezing Point Osmometer Model 3000. The obtained values are reported in Table S1.

### Reverse Transcription Reaction

To determine the RT reaction inhibition by the biopolymers, the corresponding amount of dextran (1–10%) or xanthan gum (0.001–0.1%) was added to the RT reaction mixture comprising 1× RT buffer, 5 μM RT primer (Table S2), 0.5 mM dNTP mix, 200U Maxima H minus reverse transcriptase, 20U RiboLock RNase inhibitor, and 0.135 ng of purified K562 cells total RNA. A reverse transcription reaction was performed at 42 °C for 60 min, followed by 85 °C for 5 min. The post-RT samples were diluted 50 times with nuclease-free water, and then 4 μL of diluted-cDNA was added to 40 μL of qPCR mixture comprising 1× Maxima SYBR Green/ROX qPCR master mix and 0.5 mM primer mix (Table S4) targeting *ACTB*, *TBP*, *FN1*, and *B2M* genes. The thermocycling involved initial denaturation at 95 °C for 10 min, followed by 40 cycles at 95 °C for 15 s, 60 °C for 30 s, and 72 °C for 60 s. The *C_t_* values were recorded by using software provided with the QuantStudio-1 instrument and are reported in Table S2. Reaction efficiency (2^Δ*C_t_*^) and standard deviation were calculated from three technical replicates, where Δ*C_t_* = *C_t_* (condition 1) – *C_t_* (positive control).

### PCR Inhibition

To determine the PCR inhibition by the biopolymers, the corresponding amount of dextran (1–10%) or xanthan gum (0.001–0.1%) was added to the 20 μL of PCR mixture comprising 1× Maxima SYBR Green/ROX qPCR master mix and 0.5 mM primer mix (Table S4) targeting *ACTB*, *TBP*, *FN1*, and *B2M* genes and 10 ng/μL of purified K562 cells cDNA. The samples were thermally cycled through the following program: 98 °C (10 min) for 1 cycle and 95 °C (15 s)/60 °C (30 s)/72 °C (60 s) for 40 cycles. The *C_t_* values were recorded by using software provided with the QuantStudio-1 instrument and are reported in Table S3. Reaction efficiency (2^Δ*C_t_*^) and standard deviation were calculated from three technical replicates, where Δ*C_t_* = *C_t_* (condition 1) – *C_t_* (positive control).

### Evaluation of Different DNA Polymerases

The PCR efficiency using different hot-start DNA polymerases was evaluated as follows: PCR was performed in 20 μL of reaction mix comprising 1× of Platinum Taq, Dream Taq, Maxima Taq, or KAPA polymerase master mix, 0.5 mM of pBR322 plasmid primers, 2.5 pg/μL of pBR322 plasmid, and a corresponding amount of biopolymers. Samples were subjected to thermocycling: 95 °C (5 min), followed by 30 cycles of 94 °C (30 s), 57 °C (30 s), 72 °C (1 min), and completing at 72 °C (5 min). The PCR products were visualized using agarose gel electrophoresis with a 1% agarose gel, a voltage of 150 V, 1× TAE buffer, and SYBR Green to separate and visualize the DNA fragments. Gel imaging was performed under UV light using a ChemiDoc MP system (Bio-Rad). Gel images are provided in Figure S7.

### Single-Cell RT-PCR Assay Using TaqMan Probes

The K562 cells were washed two times in an ice-cold 1× PBS and resuspended in 1× PBS with 0.05% XG or 12.5% dextran at a concentration of 5 M cells/mL. Cells were loaded into a 1 mL syringe and injected into a microfluidics device along with an RT-PCR master mix supplemented with 0.6% Igepal CA-630 and TaqMan probe targeting *RPP30* (Table S4). Droplet generation was performed using a microfluidics device having a nozzle of 30 μm depth and 30 μm width. The flow rates used were 50 μL/h for cell mix, 250 μL/h for RT-PCR reagents, and 1200 μL/h for droplet stabilization oil. After encapsulation, droplets were thermally cycled through the following program: 50 °C (5 min) for reverse transcription, 95 °C (20 s) for 1 cycle, and 95 °C (3 s)/60 °C (30 s) for 25 cycles. The fluorescence intensity of droplets was determined by imaging droplets under the epifluorescence microscope equipped with a Cy5 fluorescence filter. At least 200 droplets were analyzed in each condition using the same imaging settings (exposure time 600 ms and camera gain value 20).

## Results

### Experimental Platform to Investigate Cell Loading Process

**Figure 1:**
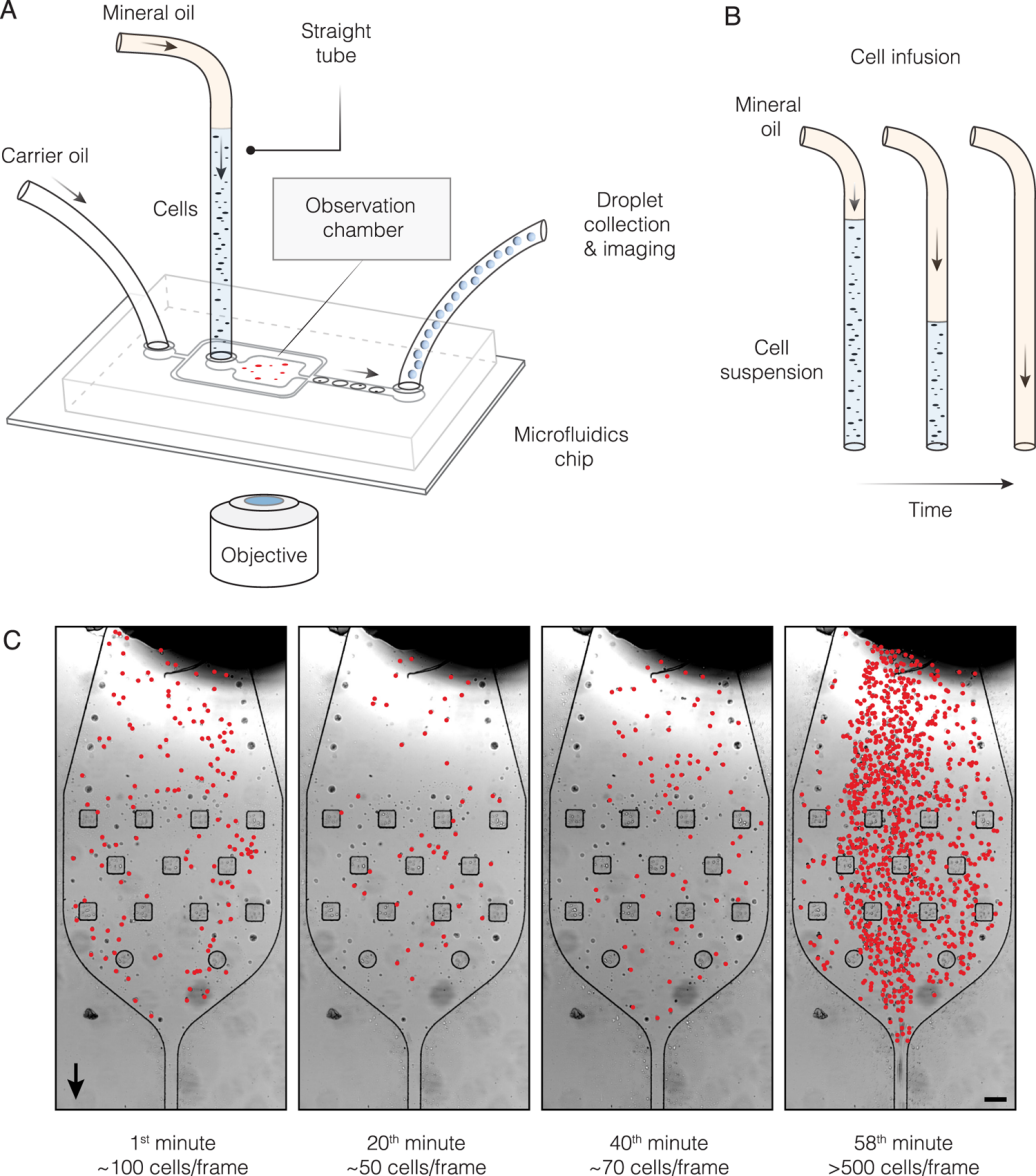
Schematics of experimental setup. (A) Schematics of the experimental platform used to investigate the cell flow dynamics. (B) Cell loading relies on water-immiscible mineral oil (orange) pushing the entire cell suspension (blue) into the microfluidics device. (C) Still digital photographs of the observation chamber at different time points with cells highlighted in red. Black arrows indicate the direction of the flow. Scale bar 100 μm.

To examine single-cell isolation biases that may occur during cell loading into microfluidics devices, we established an experimental platform schematically indicated in Figure 1 and further detailed in Figure S1. Using this platform, a suspension of cells is infused through a straight tubing along the gravitational axis with one end being inserted into the inlet of the microfluidics chip and another end connected to a syringe prefilled with mineral oil (Figure 1A). Upon infusion, the water-immiscible mineral oil pushes the cell suspension into a microfluidics device at a constant rate until the entire sample is infused (Figure 1B). The experimental conditions were set such that the entire cell suspension gets infused into a microfluidics device over the course of 60 min at a constant volumetric rate of 100 μL/h. The cells traveling through the observation chamber are then recorded by taking digital images every 30 s and counted using the machine learning algorithm ilastik^8^ (Figure 1C). Finally, the cells are encapsulated in water-in-oil droplets and collected off-chip, and resulting droplet occupancy at different time points is measured.

### Cell Loading into the Microfluidic Chip Using Standard Buffers is an Uneven Process

**Figure 2:**
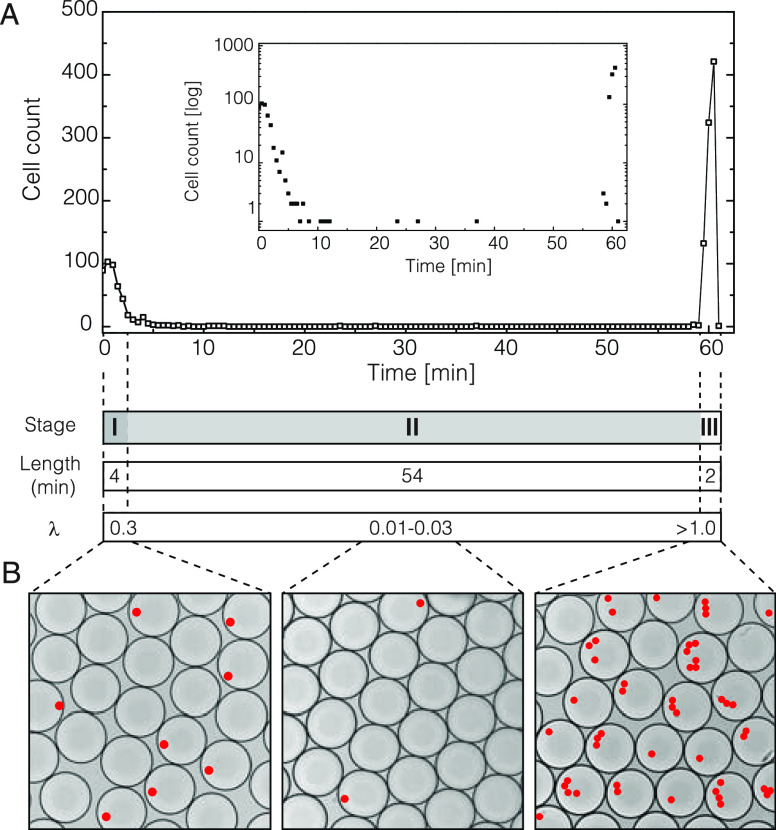
Cell flow dynamics and encapsulation in phosphate-buffered saline. The lymphoblast (K562) cells suspended in 1× PBS buffer are being continuously injected into a microfluidics device, and cells passing through the observation chamber on a microfluidics device are counted every 20 s. (A) Time trace of cells traversing the observation chamber in 1× PBS buffer (ρ_sol_ = 1.00 g/mL). The inset displays the same data but with the *Y*-axis (cell count) in a log scale. The cell flow dynamics exhibited three characteristic stages (I, II, III) that markedly differed in droplet occupancies by single cells, 0.01 > λ > 1. (B) Digital photographs of droplets collected at different time points during the experiment. The cells are highlighted in red.

We started our study by infusing cells suspended in phosphate-buffered saline (PBS) buffer and counting the cells as they traversed the observation chamber on-chip (Figure 1A). In addition, we encapsulated the cells in 1 nanoliter (nl) droplets and, at selected time intervals, assessed the droplet occupancy (λ), defined as the average number of cells per droplet. At a concentration of ∼10^6^ cells/mL, the average distance between the cells in a suspension is ∼100 μm (roughly 10× the radius of a typical cell), which translates to the expected occupancy in 1 nL droplets to be λ ≈ 1.0. However, it has long been established^6^ that due to density mismatch between the cells and the surrounding solvent, the droplet occupancy declines over time. Indeed, upon injecting the cells into the microfluidics device, we observed a rapid decay in the cell count (Figure 2A), reaching as low as 1% of the initial concentration within 3 min. Subsequently, the number of cells passing through the microfluidics chip remained low, at 1–10% of the expected values, until a final phase was reached, during which large quantities of cells traversed a microfluidics device. The three stages of cell flow dynamics on-chip were recapitulated using different cell lines (Figure S2). The droplet occupancies corresponding to each stage of the experiment also clearly reflected the irregularity of the single-cell encapsulation process: it started with an expected droplet occupancy of λ ∼ 1.0, then quickly dropped to 0.01, and surged above 1.0 by the end of encapsulation (Figure 2B). In short, these results confirmed that in common biological buffers, cell flow on-chip over time remains inconsistent and leads to a highly variable single-cell isolation process.

### Cell Loading into Microfluidics Chip Using Density-Adjusted Fluids

**Figure 3:**
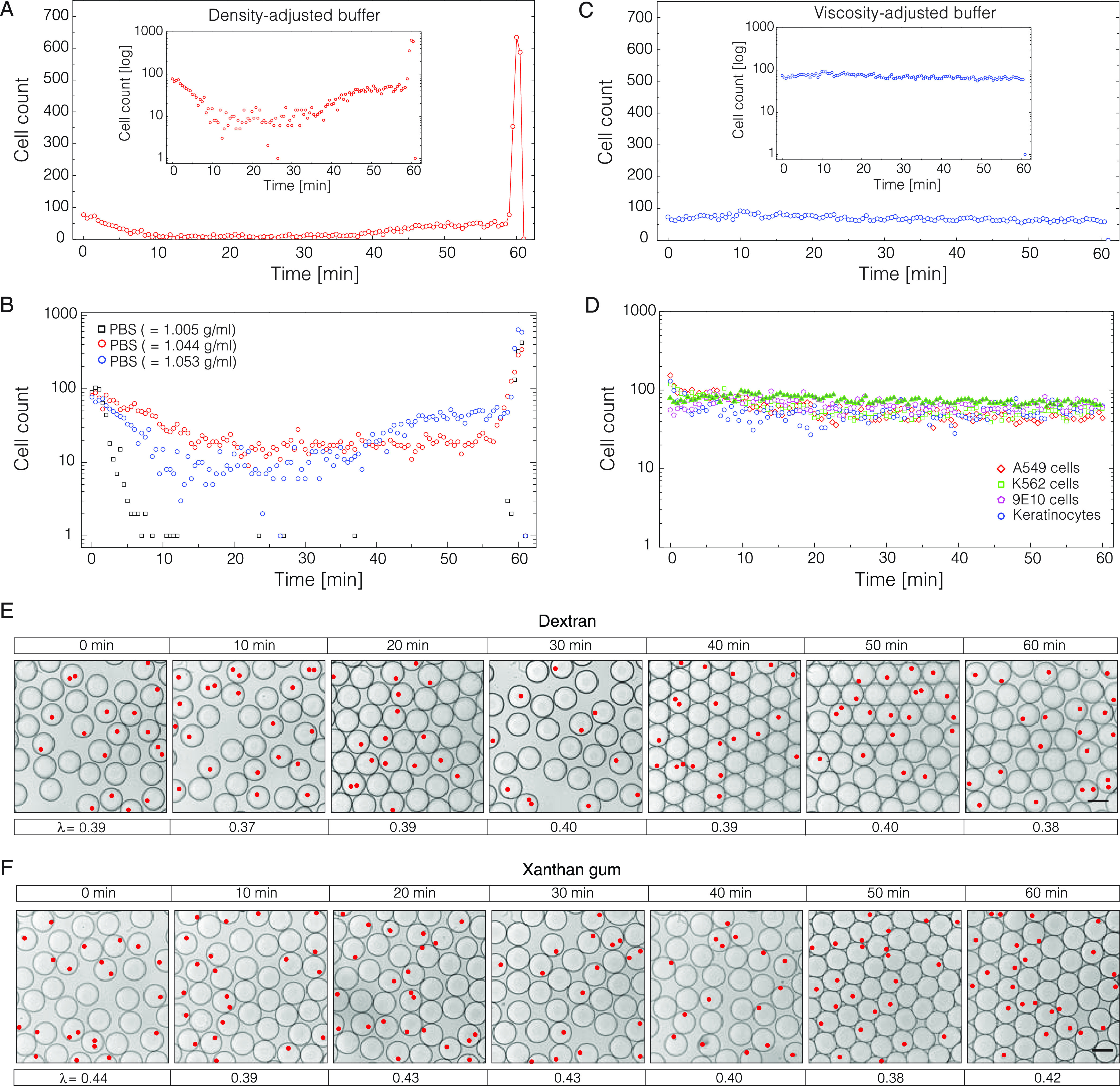
Cell flow dynamics in density-adjusted and viscosity-adjusted buffers. The time traces of lymphoblast (K562) cells are being continuously injected into the microfluidics device in density-adjusted and viscosity-adjusted buffers. (A) Cell flow dynamics in a density-adjusted buffer (ρ_sol_ = 1.053 g/mL) composed of 1× PBS and 20% Optiprep. The inset displays the same data but with the *Y*-axis (cell count) in a log scale. (B) Cell flow dynamics in PBS buffer having different density values. (C) Cell flow dynamics in a viscosity-adjusted buffer (μ_sol_ = 75 cPs) composed of 1× PBS and 15% dextran. The inset displays the same data but with the *Y*-axis (cell count) in a log scale. (D) Infusion of different types of cells in a viscosity-adjusted buffer. Solid symbols represent cell loading in 1× PBS supplemented with 15% dextran, and open symbols represent cell loading in 1× PBS supplemented with 0.05% xanthan gum. (E) Droplet occupancy over time in the presence of 15% dextran (MW 500k). (F) Droplet occupancy over time in the presence of 0.05% xanthan (MW 2000k). Scale bars: 100 μm.

To improve the cell flow dynamics, we opted to increase the density of the aqueous medium. To achieve this, we used Optiprep, a metabolically inert compound frequently used in the field of droplet microfluidics to adjust the density of the medium to that of the cells.^6^ As expected, increasing solvent density improved cell loading into the microfluidics device, yet the overall cell flow dynamics still exhibited the three characteristic stages, namely, an initial decay, followed by a sustained but reduced cell flow, and a final burst phase (Figure 3A). In contrast to regular buffer conditions, using density-adjusted PBS (ρ_sol_ = 1.053 g/mL), the first stage exhibited slower decay (*t*_1_ = 10 min vs 4 min), with the cell count dropping to 17 ± 3% (rather than 1%) of the initial cell concentration. The subsequent phase, lasting for 50 min, featured a consistent but highly diminished number of cells flowing through the chip, followed by a gradual recovery up to 40–50% of the expected cell count. A final burst phase lasted for approximately 2 min and accounted for roughly two-thirds of all injected cells. Similar cell flow dynamics was also recapitulated when injecting a different type of cells (Figure S3). Based on these results, we concluded that the use of cell-density-adjusted solvent only modestly improves the uniformity and consistency of cell loading. Specifically, even in the most promising conditions (ρ_sol_ = 1.044 g/mL), the coefficient of variation (CV) remained notably high (>100%) (Figure 3B).

### Higher Viscosity Fluids Improve the Consistency of Cell Loading and Encapsulation

To find optimal conditions for consistent and sustained cell flow, we postulated that increasing the viscosity of the aqueous medium, rather than altering its density, would effectively suppress cell sedimentation and, consequently, lead to improved cell loading and encapsulation. Our notion stems from Stoke’s velocity relationship (see Supporting Information, Note S1), which suggests that by increasing the viscosity of the solvent, the cell velocity should approach zero, thereby preventing cells from sedimentation, or rising up, along the gravitational axis. To test this, we sought polymers that are biocompatible and chemically neutral as well as ensured that they do not exert a significant osmotic pressure on cells or undergo phase separation in the presence of salts. We selected three high-molecular-weight biopolymers (≥100 kDa) belonging to the polysaccharide class, namely, dextran, xanthan gum (XG), and methylcellulose (see the Material and Methods section). We spiked selected biopolymers to cell suspension to obtain fluid viscosities ranging from 6.6 to 85 cPs, injected the cells into the microfluidics device, and recorded the number of cells passing through the observation chamber every 10 s over the span of 20 min. We determined the sedimentation rate of cells under each tested condition by applying the exponential decay function. As anticipated, with increasing fluid viscosity and irrespectively of the biopolymers used, the sedimentation rate approached zero (Figure S4). The nearly complete arrest of cell sedimentation was achieved when the viscosity of the Newtonian fluids reached the range of approximately 40–50 cPs, even though the solution’s density increased only marginally. In the case of the non-Newtonian fluid, achieving the same level of cell sedimentation arrest appeared to necessitate higher viscosity (μ ≈ 85 cPs), although the exact viscosity values are sensitive to shear stress and are challenging to estimate within our experimental settings. Subsequently, we chose one of the conditions that exhibited the lowest sedimentation rates, specifically, 1× PBS buffer comprising 15% (w/v) 500 kDa dextran and having a viscosity of approximately 75 cPs. We prepared cell suspension (∼10^5^ cells/100 μL) and injected it into a microfluidics device over the course of 60 min (Figure 3C). Noticeably, the variability of the cell flow remained low over the entire course of the experiment (CV = 4.5%). In comparison, cell density-adjusted buffers with low viscosities (μ = 1.4 cPs) failed to achieve the same level of consistency and exhibited very broad cell flow variability (Figure 3B), with CV ≈ 80% (excluding the burst phase) and CV ≈ 200% (including the burst phase).

In a separate set of experiments, we also confirmed that in a viscosity-adjusted buffer, different types of cells exhibit reduced sedimentation and display steady flow dynamics (Figure 3D) and low single-cell encapsulation variability (Figure 3E,F). Therefore, our results prove that aqueous solvents having increased viscosity, rather than cell-adjusted density, provide a more reliable approach to mitigate cell sedimentation and as a result are better suited for achieving consistent and unbiased single-cell isolation.

To examine whether the fluid viscosity that was sufficient to arrest cell sedimentation could hinder droplet generation, we monitored droplet formation on-chip with a high-speed camera (Fastec HS7) at a fixed flow rate ratio (100 μL/for the aqueous phase and 300 μL/h for the carrier oil). We found that for dextran solutions at viscosities as high as 100 cPs, water-in-oil droplet generation remains stable over extended periods of time, while at 200 cPs droplet generation resorts to jetting (Figure S5A). In contrast, droplet generation using solutions with XG remained uniform and stable even when exceeding 200 cPs (Figure S5B).

### Compatibility of Biopolymers for Cell-Based and Enzymatic Assays

Certain types of biopolymers are known to have adverse effects on cell-based and enzymatic assays. For example, some polymers may increase hyperosmotic pressure,^9^ induce undesirable cell clumping,^10,11^ or inhibit enzymatic reactions such as the nucleic acid analysis by PCR.^12^ Therefore, we evaluated whether the biopolymers used in our study could negatively impact cells or enzymatic reactions. Initially, we measured the osmolarity of biopolymer solutions and found that at the highest concentrations used in this work, there is only a minor (up to 10%) increase in the solution osmolarity (Table S1). Furthermore, using the dextran biopolymer, we observed insignificant cell aggregation (clumping) at all but 5% solution (Figure S6A), while in 0.05% XG, the cell aggregation was completely absent with four different cell lines tested (Figure S6B).

**Figure 4:**
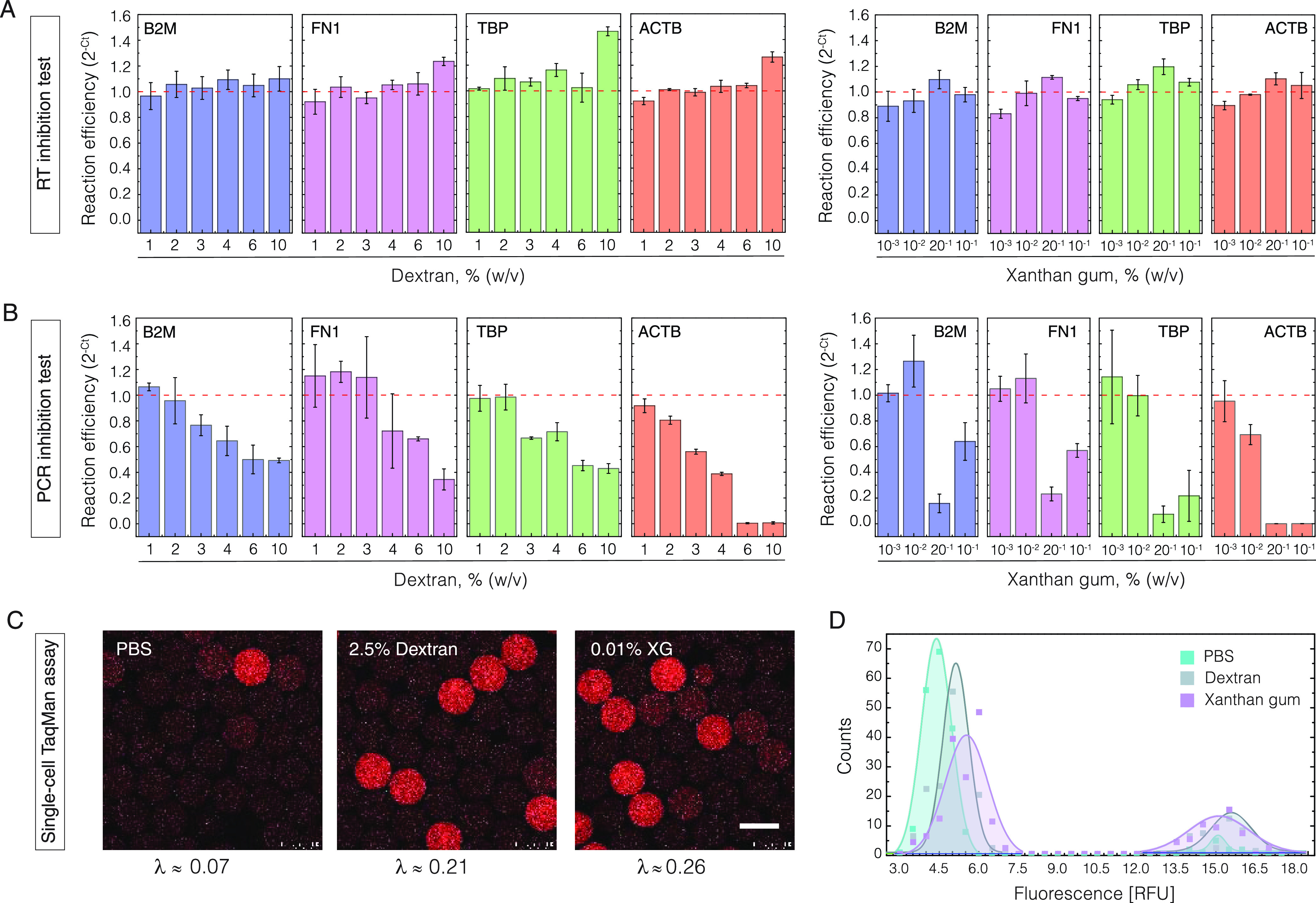
Evaluation of nucleic acid amplification and analysis in the presence of biopolymers. (A) Reverse transcription (RT) reaction in the presence of dextran (left) and xanthan gum (right). The column height and error bars indicate the mean reaction efficiency and standard deviation, respectively (*n* = 3). (B) Polymerase chain reaction (PCR) in the presence of dextran (left) and xanthan gum (right). The column height and error bars indicate the mean reaction efficiency and standard deviation, respectively (*n* = 3). (C) Droplet-based single-cell RT-PCR assay using a TaqMan probe in the absence or presence of biopolymers. The percentages indicate the final biopolymer fraction in droplets. The scale bar denotes 100 μm. (D) Fluorescence distribution of droplets after single-cell RT-PCR using TaqMan probes. The population displaying high fluorescence values (>12 RFU) are the droplets with cells, while the population having low fluorescence values (<8 RFU) are empty droplets. Note the reduced count of positive droplets in the absence of biopolymers.

Finally, to understand whether the presence of dextran or XG biopolymers might inhibit the nucleic acid analysis, we assessed PCR and RT reaction efficiency by conducting qPCR (real-time PCR) on a cDNA template as well as two-step RT-qPCR (reverse transcription and real-time PCR) on a total RNA template. The degree of inhibition was estimated by the 2^–Δ*C_t_*^ method, targeting four genes (ACTB, B2M, FN1, and TBP) in the presence of varying amounts of biopolymers. The results presented in Figure 4 indicate that the cDNA synthesis is far less sensitive to biopolymer concentrations than cDNA amplification by PCR. Even at 10% dextran or 0.05% XG, the RT reaction yields were similar to or higher than those of the control sample (Figure 4A). Contrarily, the amount of PCR product dropped significantly when the biopolymer fraction in a reaction mix reached ∼3% for dextran and 0.05% for XG (Figure 4B). Among the four hot-start PCR enzymes tested in this study, the DNA polymerases under the brand names KAPA HiFi HotStart (Roche) and Phire Tissue Direct (Thermo Fisher Scientific) appeared to show the lowest degree of inhibition (Figure S7). Importantly, irrespective of the enzyme tested, the PCR specificity remained high, as confirmed by monophasic melting transitions and the size of PCR amplicons (Figures S7 and S8). Based on these results, we concluded that nucleic acid amplification and analysis of single cells can be conducted in the presence of dextran or XG biopolymers without corrupting the reaction specificity.

To confirm this notion, we performed a one-step RT-PCR assay on single cells using TaqMan probes. We prepared K562 cells in 1× PBS comprising either 0.05% XG or 12.5% dextran and loaded them in 1 nL volume droplets (at λ ≈ 0.2–0.3) along with the TaqMan assay reagents targeting RPP30 transcripts. We adjusted the flow rates of the aqueous phases 1:5 such that the XG and dextran amounts in a droplet would end up being 0.01 and 2.0%, respectively. As expected, following RT-PCR, the droplets carrying cells turned red due to the cleavage of TaqMan probes and the release of the reporter dye (Figure 4C). The fluorescence signal intensity of cell-containing droplets was comparable between different samples and was ∼3-fold higher than that of droplets having no cells (Figure 4D). As such, the scRT-PCR results serve as an additional confirmation that in the presence of low amounts of dextran or XG biopolymers, the nucleic acid amplification and analysis of single cells do not introduce adverse effects. The droplet occupancy of samples prepared with the biopolymers matched closely the theoretical expectations based on Poisson distribution, yet the sample lacking these additives displayed a significant reduction of positive droplets, thus indicative of cell loss due to sedimentation. Overall, the results presented in this work may benefit various biological methods such as scRNA-Seq or scDNA-Seq that rely on unbiased single-cell isolation and analysis.

## Discussion

A growing number of biological and biomedical applications rely on microfluidic tools for isolating and characterizing the phenotype and genotype of individual cells. However, a critical step in these efforts, unbiased and uniform single-cell isolation, has been largely neglected and overlooked. In this work, we investigated cell loading into a microfluidic chip and droplets over an extended period of time (60 min) and found that the standard microfluidic setup displays large variability and inconsistency. We found that cell loading into a microfluidics device displays three characteristic stages that can be reproduced across different cell types. Specifically, in regular aqueous buffers such as phosphate-buffered saline (1× PBS, ρsol ∼1.00 g/mL), cells sediment fast, and as a result, the number of cells entering the chip (and droplets) decays rapidly over time to only 1–3% of the expected cell count. The cell flow at this reduced level persists throughout the remaining course of the experiment until the last few minutes, during which a large fraction of all cells (∼2/3rds of all cells) gets injected into the chip at a constant rate. Adjusting medium density to that of the cells (ρ_sol_ = 1.044–1.053 g/mL) somewhat improves the consistency of cell loading into a chip, yet cell flow dynamics remains highly variable with CV ≈ 16–80%. These results point out the potential single-cell isolation biases that are likely to arise when attempting to analyze diverse and heterogeneous populations since there will always be a fraction of cells that will be either lighter, or heavier, than the density-adjusted solvent in which cells are dispersed.

We show that increasing fluid viscosity in cell suspensions provides a simple and efficient way to stabilize cell loading into a microfluidics device and achieve consistent single-cell isolation in water-in-oil droplets over extended periods of time. We achieve this by supplementing cell suspensions with high-molecular weight biopolymers. For instance, 0.75% methylcellulose (∼86 kDa) solution having a viscosity of 44 cPs nearly halts cell sedimentation, even though the solution’s density is increased only by a few decimals (from 1.005 to 1.015 g/mL). In contrast, maintaining the same solution density (1.015 g/mL) without adjusting the viscosity results in almost complete cell sedimentation within 20 min. Furthermore, adjusting the solution density alone (without modifying the viscosity) does not lead to stable and consistent cell loading (Figure 3B). Only when the solution viscosity is increased does the cell flow into a chip become steady and uniform over the entire course of the experiment (Figure 3D), with highly consistent single-cell encapsulation (Figure 3E,F).

Indeed, not all biopolymers will be suitable for preventing cells from sedimentation, as some are known to cause undesirable cell clumping,^10^ electrostatic association with the cell membrane,^13,14^ or inhibition of enzymatic assays.^12^ In our study, we used high-molecular-weight polysaccharides (i.e., dextran and xanthan gum) that do not exert significant osmotic pressure change on the cells nor cell aggregation or clumping. In addition, these biopolymers were compatible with nucleic acid amplification and analysis (Figure 4). As such, the approach suggested in this work should improve the reproducibility of single-cell genomic applications by making single-cell isolation more accurate and uniform. For example, it is well-known that the cell capture using droplet-based systems such as chromium (10× Genomics) often results in the underrepresentation of certain cell types.^3^ While poor cell recovery can be partly attributed to biological effects such as fragility of the cells and premature lysis, considering that the cell encapsulation is typically performed in standard buffers (i.e., 1× PBS), increasing fluid viscosity holds promise to improve single-cell capture efficiencies. Indeed, the results of this work may have practical implications beyond microfluidic systems. Cell sedimentation is a common hurdle for FACS, three-dimensional (3D) printing, and other techniques that rely on single-cell isolation and analysis. Our results provide a practical solution to circumvent undesirable cell losses due to sedimentation and overcome single-cell isolation biases in microfluidics.

## Supporting Information Available

Description of the experimental setup including theoretical background for cell sedimentation (Note S1), the osmotic pressure of biopolymer solution (Table S1), the time trace of cell flow in density- and viscosity-adjusted buffers, evaluation of nucleic acid amplification inhibition (Tables S2 and S3), the signal intensity of single-cell TaqMan assay (Table S4), and DNA primers used in the study (Table S5) and Figures S1–S8 (PDF)
