# Supplementary material for: Increasing Fluid Viscosity Ensures Consistent Single-Cell Encapsulation
Source: Anal Chem. 2024 Apr 22;96(18):6898–905. doi: 10.1021/acs.analchem.3c05243 (PMC11079858; doi:10.1021/acs.analchem.3c05243)
Supplement: Supplementary file 1 — ac3c05243_si_001.pdf [file ac3c05243_si_001.pdf]

SUPPORTING INFORMATION

Increasing fluid viscosity ensures consistent single cell encapsulation

Emilė Pranauskaitė, Valdemaras Milkus, Justas Ritmejeris, Rapolas Žilionis, Linas Mažutis*

Institute of Biotechnology, Life Sciences Centre, Vilnius University, 7 Saulėtekio av., Vilnius, Lithuania

* Corresponding author: linas.mazutis@bti.vu.lt

Table of Contents:

Theoretical background for cell sedimentation …..……..……………..…………. P. S2

Supplementary Tables ………………..………………...……………..………...… P. S3

Experiment setup ………………..……………..……………….……………….. P. S5

The time trace of cell flow in 1x PBS buffer ………..……………….………..… P. S6

The time trace of cell flow in a cell-density adjusted PBS buffer ……………....... P. S7

Cell sedimentation rate in viscosity-adjusted PBS buffer ...………….……..…….. P. S8

Droplet generation with viscous fluids ..……………..……………..………...… P. S9

Cell clumping over time in a PBS buffer supplemented with dextran and Xanthan gum biopolymers ……………..……………..……………..……………..…………..… P. S10

Inhibition test of PCR enzymes by dextran and Xanthan gum ………………….. P. S11

The specificity of RT and PCR in the presence of dextran and Xanthan gum …. P. S12

Supplementary Note 1

Theoretical background for cell sedimentation

Cells suspended in a stationary fluid experience the forces of gravity, the Archimedes (buoyancy) force and the viscous drag force. The sum of the action of these forces defines the magnitude and the direction of the cell velocity. Under the effect of gravity, $F_g = m_c g = \rho_c V_c g$ the axis of motion is directed downwards. Here, ρ_c represents the average density of the cell, V_c is the cell volume and g is the gravitational acceleration. The forces opposing the motion of the cell along the gravity axis (sedimentation) are the Archimedes force $F_A = \rho_f g V_c$ and the viscous drag force $F_v = 6\pi\eta R_c v$, where ρ_f is the fluid density, R_c is the cell radius and v is the velocity of a cell. The cell motion m_c can be expressed as the sum of opposing forces:

$$m_c \frac{dv}{dt} = \rho_c V_c g - \rho_f g V_c - 6\pi\eta R_c v = gV_c(\rho_c - \rho_f) - 6\pi\eta R_c v \quad \text{(S1)}$$

The integral of equation (1) can be expressed as:

$$\int_0^v \frac{dv}{\frac{gV_c(\rho_c-\rho_f)}{6\pi\eta R_c} - v} = \int_0^t \frac{6\pi\eta R_c}{m_c} dt \quad \text{(S2)}$$

leading to the solution, where the cell velocity, v is expressed as a time-dependent function:

$$v = \frac{gV_c(\rho_c-\rho_f)}{6\pi\eta R_c}\left(1 - exp\left[-\frac{6\pi\eta R_c t}{m_c}\right]\right) = \frac{2gR_c^2}{9\eta}(\rho_c - \rho_f)\left(1 - exp\left[-\frac{9\eta t}{2\rho_c R_c^2}\right]\right) \quad \text{(S3)}$$

For microscale objects in the order 10^{-6} m (e.g. cells), the exponential function is decaying fast and thus can be omitted leading to Stokes' velocity

$$v_{St} = \frac{2gR_c^2}{9\eta}(\rho_c - \rho_f). \quad \text{(S4)}$$

Supplementary Tables

Supplementary Table S1. Osmotic pressure of biopolymers in phosphate-buffered saline solution

Solution*	Osmotic pressure (mOsmol/kg), Mean ± stdev, n = 2
1x PBS (Phosphate Buffered Saline [pH 7.4])	298 ± 2.1
5% Dextran 500k in 1x PBS	295 ± 0.7
10% Dextran 500k in 1x PBS	310 ± 2.8
15% Dextran 500k in 1x PBS	333 ± 1.4
0.5% Methylcellulose in 1x PBS	330 ± 1.4
0.75% Methylcellulose in 1x PBS	338 ± 1.0
0.025% Xanthan in 1x PBS	315 ± 1.4
0.05% Xanthan in 1x PBS	315 ± 1.0

* % are given in (w/v).

Supplementary Table S2. The Ct values of the marker genes determined by RT-qPCR

Sample	The Ct values* of marker genes			
	ACTB	B2M	FN1	TBP
Reference	20.91 ± 0.006	21.72 ± 0.1	28.65 ± 0.07	26.05 ± 0.05
1% Dex	21.03 ± 0.04	21.78 ± 0.03	28.77 ± 0.08	26.08 ± 0.03
2% Dex	20.90 ± 0.005	21.65 ± 0.02	28.60 ± 0.04	25.93 ± 0.08
3% Dex	20.93 ± 0.04	21.68 ± 0.02	28.72 ± 0.06	25.94 ± 0.02
4% Dex	20.86 ± 0.07	21.59 ± 0.04	28.57 ± 0.08	25.84 ± 0.06
6% Dex	20.85 ± 0.02	21.66 ± 0.007	28.56 ± 0.05	25.98 ± 0.09
10% Dex	20.57 ± 0.04	21.59 ± 0.1	28.34 ± 0.05	25.51 ± 0.04
0.001% XG	21.07 ± 0.06	21.90 ± 0.07	28.91 ± 0.07	26.14 ± 0.01
0.01% XG	20.94 ± 0.01	21.90 ± 0.06	28.66 ± 0.09	25.98 ± 0.006
0.05% XG	20.77 ± 0.07	21.59 ± 0.04	28.49 ± 0.09	25.80 ± 0.04
0.1% XG	20.84 ± 0.1	21.75 ± 0.05	28.72 ± 0.09	25.95 ± 0.05
NTC	No signal	No signal	No signal	No signal

* The Ct values were determined by real-time qPCR on QuantStudio 1 instrument using 40 ng/µl of total RNA extracted from K-562.The primer pairs used for qPCR are listed in Supplementary Table S4. The values represent the mean and standard deviation from three technical replicates.

Supplementary Table S3. The Ct values of the marker genes determined by RT-qPCR

Sample	The Ct values* of marker genes			
	ACTB	B2M	FN1	TBP
Reference	17.48 ± 0.06	17.86 ± 0.1	24.35 ± 0.2	23.95 ± 0.04
1% Dex	17.5 ± 0.05	17.77 ± 0.1	24.16 ± 0.1	23.98 ± 0.1
2% Dex	17.77 ± 0.4	17.95 ± 0.2	24.11 ± 0.1	23.98 ± 0.1
3% Dex	18.29 ± 0.05	18.25 ± 0.2	24.20 ± 0.2	24.53 ± 0.07
4% Dex	18.82 ± 0.01	18.51 ± 0.3	24.90 ± 0.4	24.44 ± 0.1
6% Dex	26.42 ± 1.9	18.89 ± 0.2	24.94 ± 0.2	25.12 ± 0.1
10% Dex	26.35 ± 2.7	18.89 ± 0.2	25.91 ± 0.2	25.18 ± 0.1
0.001% XG	17.57 ± 0.2	17.84 ± 0.01	24.28 ± 0.07	23.81 ± 0.4
0.01% XG	18.02 ± 0.2	17.54 ± 0.3	24.18 ± 0.2	23.97 ± 0.2
0.05% XG	35.34 ± 1.2	20.62 ± 0.7	26.48 ± 0.3	31.39 ± 7.4
0.1% XG	28.05 ± 0.8	18.53 ± 0.3	25.16 ± 0.07	29.60 ± 6.9
NTC	No signal	No signal	No signal	No signal

Supplementary Table S4. Mean and sigma values of single-cell TaqMan assay

Sample	Negative droplets		Positive droplets	
	Mean	Sigma	Mean	Sigma
Reference (PBS)	4186.7	1053.56	14897.6	384.8
Dextran	5750	849.3	15250	1062.5
Xanthan gum	5289.8	1501.4	14867	1663.3

Supplementary Table S5. DNA primers used in the study

Primer name	Sequence
**RPP30_F**	CTGACCTGAAGGCTGACGCCGGACTTGTGGAGACAGC
**RPP30_R**	GGTTTTTCAATTTCCTGTTTCTTTTCCTTAAAGTCAACG
**TaqMan probe**	JUN-TTCTGACCTGAAGGCTCTGCGC-QSY
**ACTB-F**	CGCCGCCAGCTCACC
**ACTB-R**	TCTCCATGTCGTCCCAGTTG
**B2M-F**	CTCACGTCATCCAGCAGAGAA
**B2M-R**	TGCTTACATGTCTCGATCCCAC
**FN1-F**	ACAAACACTAATGTTAATTGCCCA
**FN1-R**	AACTCCCAGGGTGATGCTTG
**TBP-F**	TTTGCAGTGACCCAGCATC
**TBP-R**	AGAAACCCTTGCGCTGGAA
**RT primer InDrop-T30-trim3**	CTACACGACGCTCTTCCGATCTNNNNNNTTTTTTTTTTTTTTTTTTTTTTTTTTTTTT

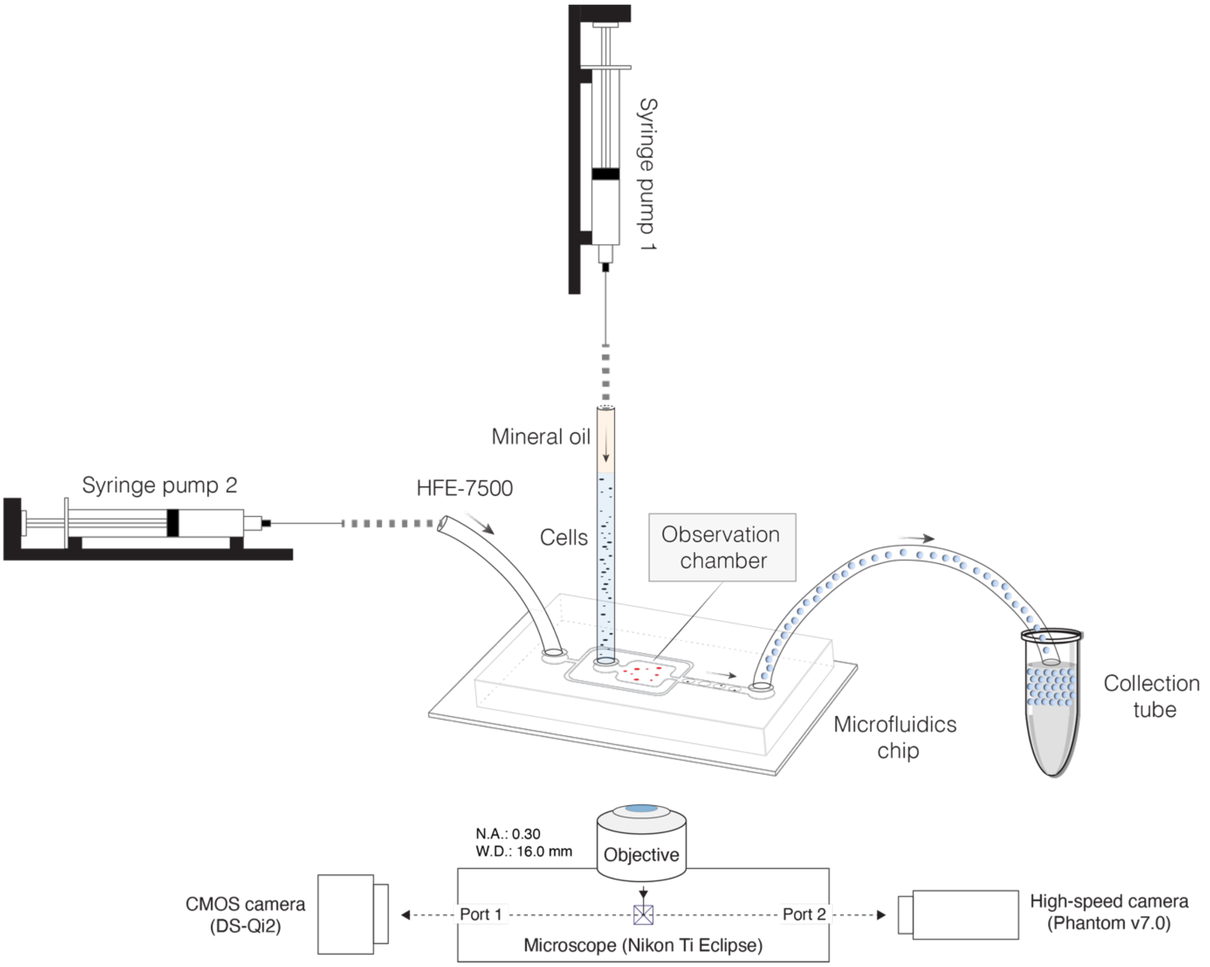

Figure S1. Experiment setup. Schematics of experimental platform. The microfluidics device is connected to two syringe pumps for infusion of cell suspension (Syringe pump 1) and for infusion of immiscible carrier oil (Syringe pump 2). The syringe placed on Pump 1 is filled with mineral oil, which upon infusion, pushes the cell suspension into a microfluidics device until the entire sample is consumed. The cells passing through a microfluidics device (observation chamber) are recorded by capturing the digital images every 30 seconds using a CMOS camera (DS-Qi2). The syringe on Pump 2 is filled with HFE7500 carrier oil supplemented with droplet-stabilizing fluorosurfactant. The water-in-oil droplets are generated at a flow-focusing junction and recorded using a high-speed camera (Phantom v7 or HiSpec HS7), collected into a tube, and further inspected under the bright field microscope to estimate droplet occupancy by single cells.

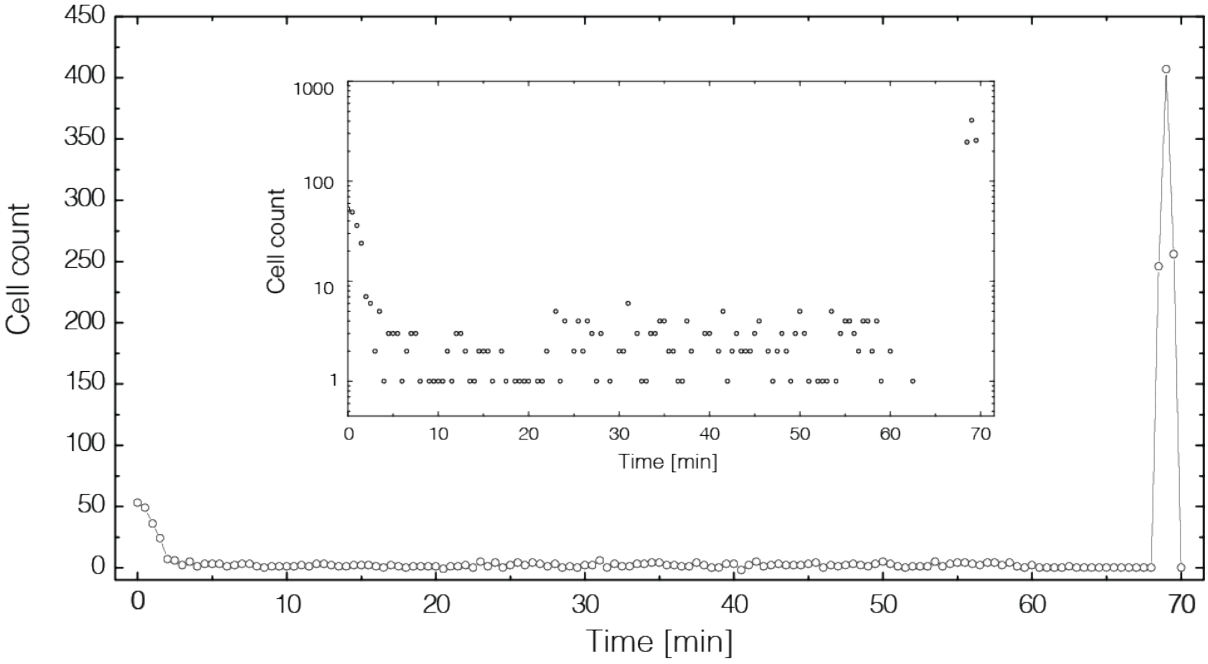

Figure S2. The time trace of cell flow in 1x PBS buffer. The hybridoma (9e10) cells are being continuously injected into a microfluidics device over the course of 70 min and cell number passing the observation chamber is recorded. Note how the initial cell count passing through the microfluidics device drops down to ~1% in 3 minutes and then continues to fluctuate within the 0-10% range until the burst at 68th minute, during which 2/3rds of all cells pass through the device. The inset displays the same data but with Y-axis (Cell count) in a log scale.

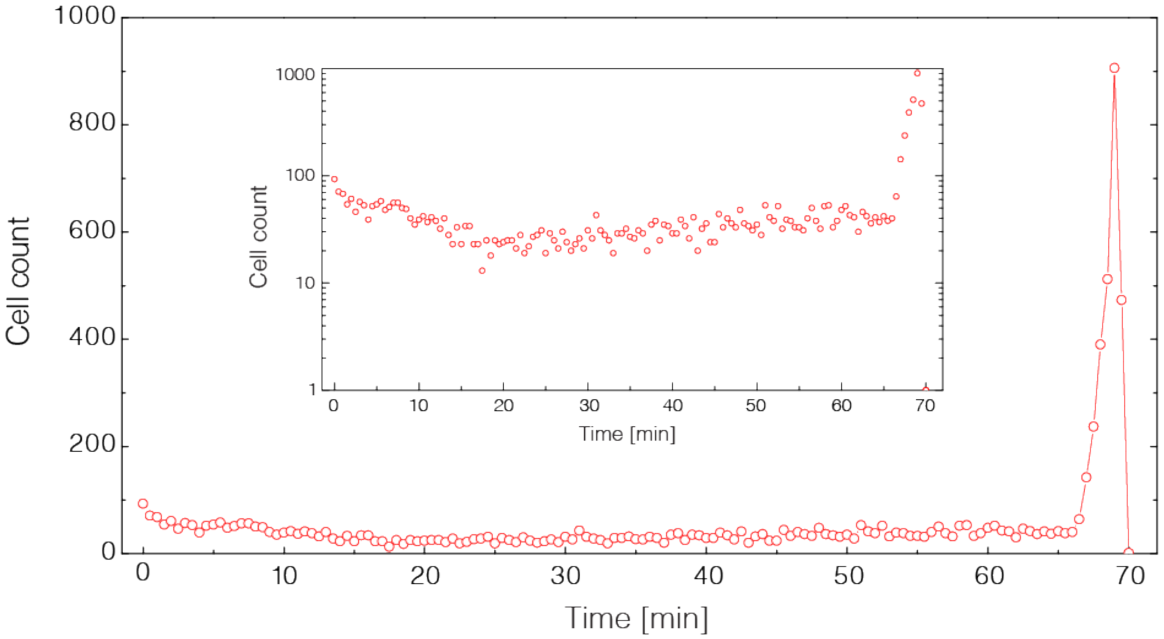

Figure S3. The time trace of cell flow in a cell-density adjusted solution. The hybridoma (9e10) cells are being continuously injected into a microfluidics device in the presence of 20% Optiprep (ρ_{sol} = 1.053 g/ml) over the course of 70 min. Noticeably, the use of 1x PBS buffer with 20% Optiprep improves cell loading as compared to 1x PBS (Figure S2). The initial number of cells passing through the microfluidics device drops down to ~20% in 15 minutes and then slowly recovers to approx. 30-40% of the initial count until the final burst during which ~2/3^{rds} of all cells pass through the device. The inset displays the same data but with Y-axis (Cell count) in a log scale.

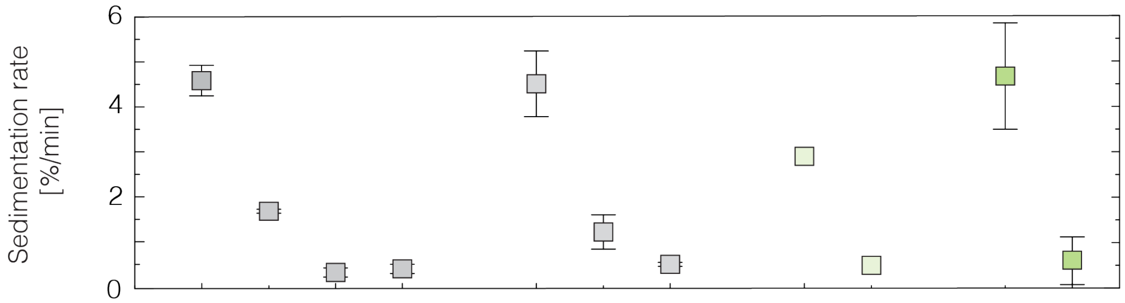

Amount (%, w/v)	5	10	12.5	15	5	10	15	0.5	0.75	0.01	0.05
Density (g/ml)	1.018	1.034	1.045	1.056	1.018	1.034	1.056	1.010	1.015	1.02	1.03
Viscosity (cPs)	6.6	24	45	75	6.6	24	75	17	44	~43*	~85*
Cell line	K562				9e10			K562		K562	
Biopolymer	Dextran				Dextran			MetCellulose		Xanthan gum	
Fluid	Newtonian				Newtonian			Newtonian		Non-Newtonian	

Figure S4. Cell sedimentation rate in viscosity-adjusted PBS buffer. The lymphoblast cells (K562) were suspended in 1x PBS buffer supplemented with varying amounts of biopolymers and injected into a microfluidics device using the experimental setup indicated in Figure S1. The cells passing the observation chamber were counted and the sedimentation rate was extracted from the exponential decay function. Note, that the buffer in which cells are suspended undergoes continuous injection by the syringe pump, therefore sedimentation rate involves two parameters, passive cell sedimentation and injection along the gravitational axis.

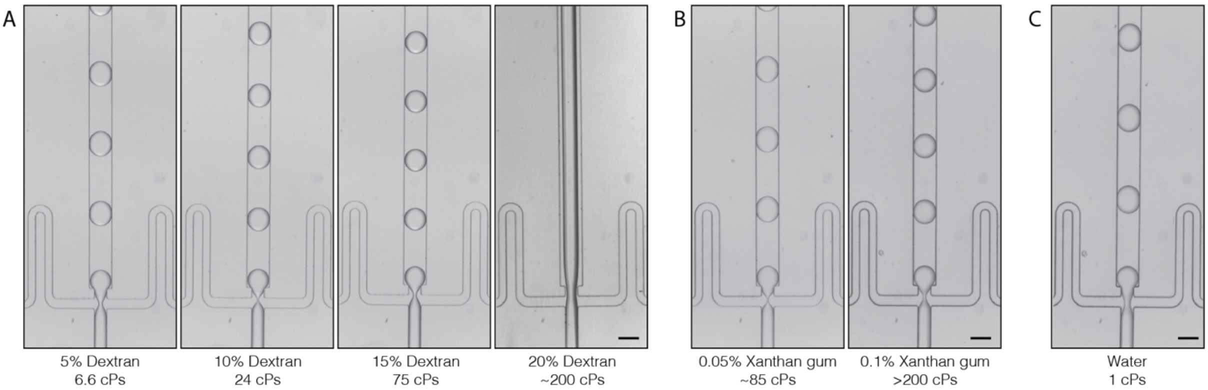

Figure S5. Droplet generation with viscous fluids. Phosphate buffered saline (1x PBS) was supplemented with increasing amounts of Dextran or Xanthan gum and emulsified on an 80 µm deep microfluidics device, using flow rates at 100 µl/hr for aqueous phase and 300 µl/fr for droplet stabilization oil. The droplet generation was followed for 60 min. Droplet generation in the presence of dextran (A), xanthan gum (B), or no additive (C). Scale bars, 100 µm.

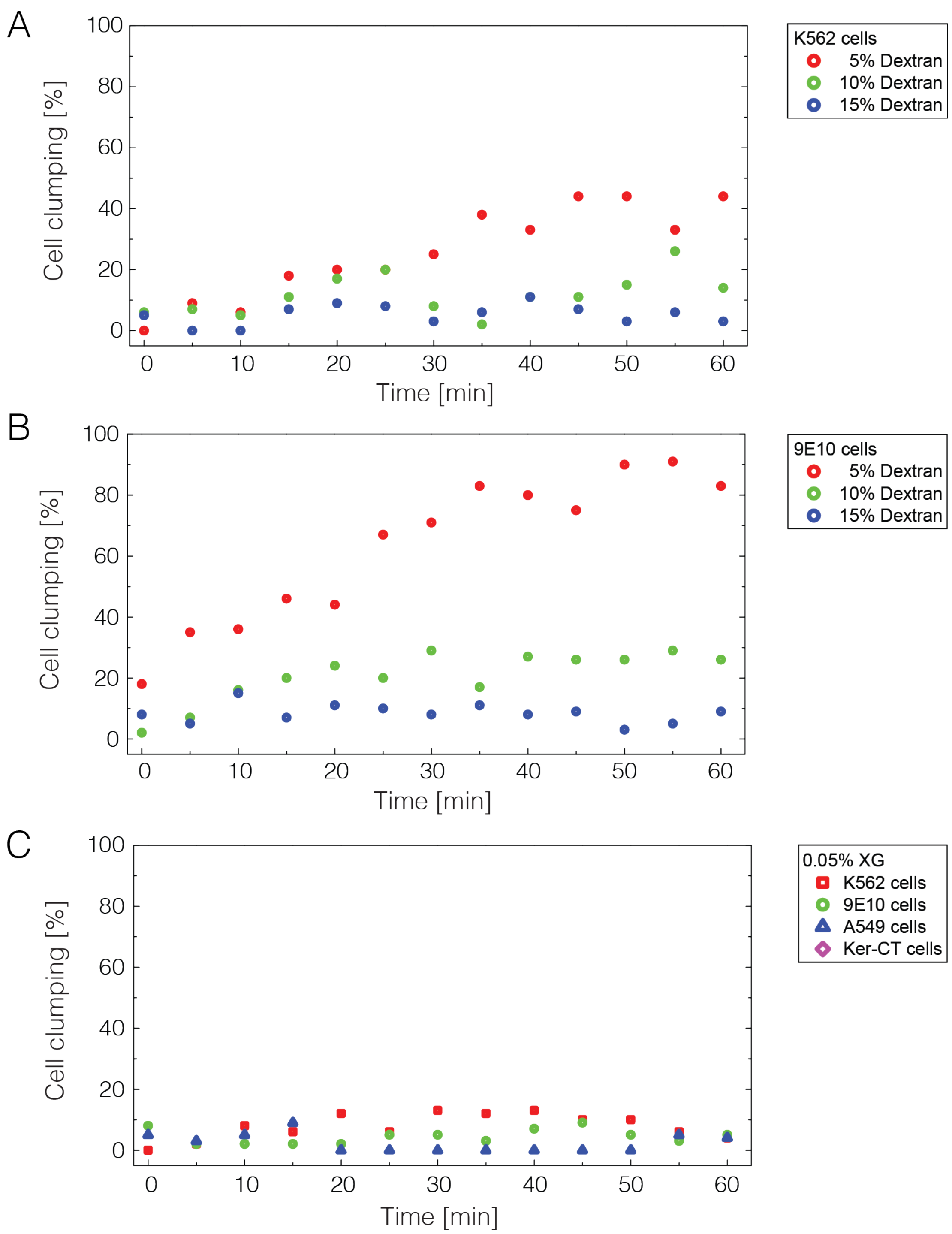

Figure S6. Cell clumping over time in a PBS buffer supplemented with dextran and Xanthan gum. Cell clumping is defined as the number of aggregated cells (n ≥ 2) over the total number of cells. The cell counting was conducted every 5 minutes as the cells traversed the microfluidics device using the experimental setup indicated in Figure S1. **A)** Clumping of suspension (K-562) cells over the course of 60 minutes in 1x PBS supplemented with 5, 10, or 15% dextran. **B)** Clumping of semi-adherent (9E10) cells over the course of 60 minutes in 1x PBS supplemented with 5, 10, or 15% dextran. **C)** Clumping of different types of cells in 1x PBS supplemented with 0.05% Xanthan gum.

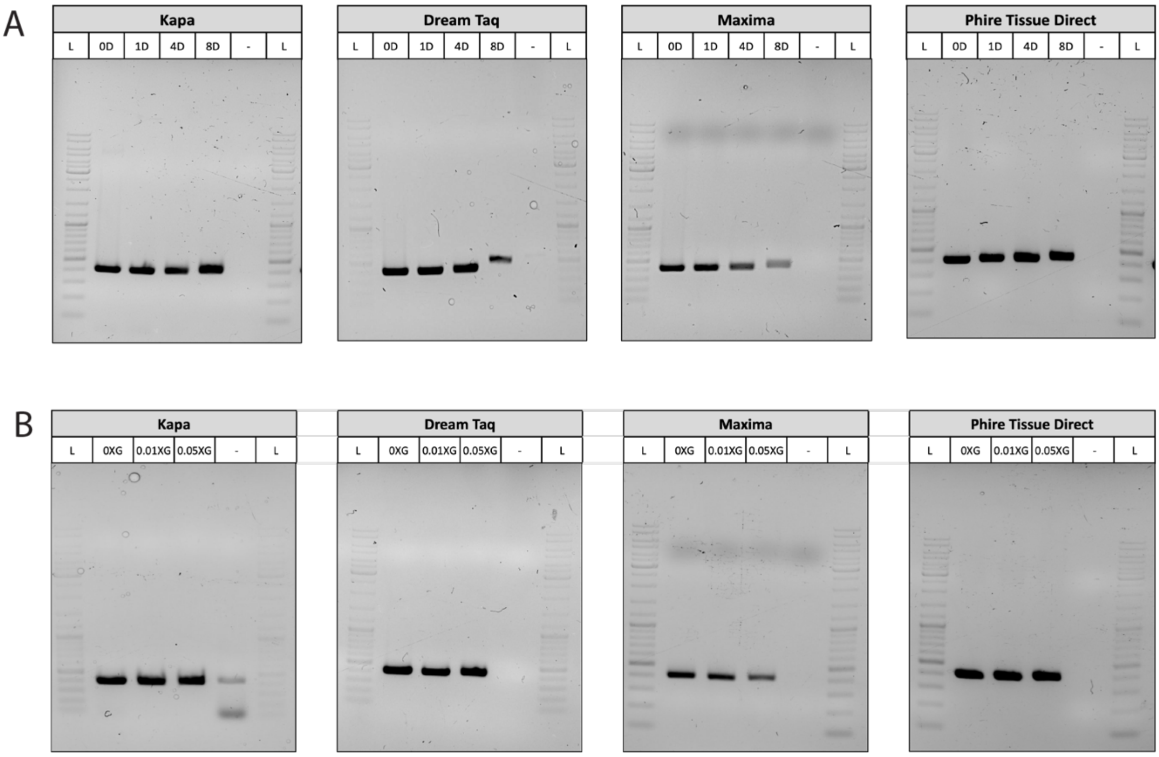

Figure S7. Inhibition test of PCR enzymes by dextran and Xanthan gum. The DNA amplification by four different PCR enzymes, KAPA (Roche, cat. no. KK2602), DreamTaq (Thermo Fisher Scientific, cat. no. K9011), Maxima (Thermo Fisher Scientific, cat. no. K0222) and Phire Tissue Direct (Thermo Fisher Scientific, cat. no. F170S) in the presence of dextran or Xanthan gum biopolymer. **A)** DNA amplification by PCR in the presence of dextran. **B**) DNA amplification by PCR in the presence of Xanthan gum. L – ladder (ThermoFisher Scientific, SM0331), 0D, 1D, 4D and 8D indicates dextran fraction (%, [w/v]), whereas 0XG, 0.01XG and 0.05XG indicated Xanthan gum fraction (%, [w/v]). The minus sign indicates no template control. Note, at each condition tested the PCR produced a single specific amplicon band (400 bp), indicating that the presence of biopolymers does not impact reaction specificity.

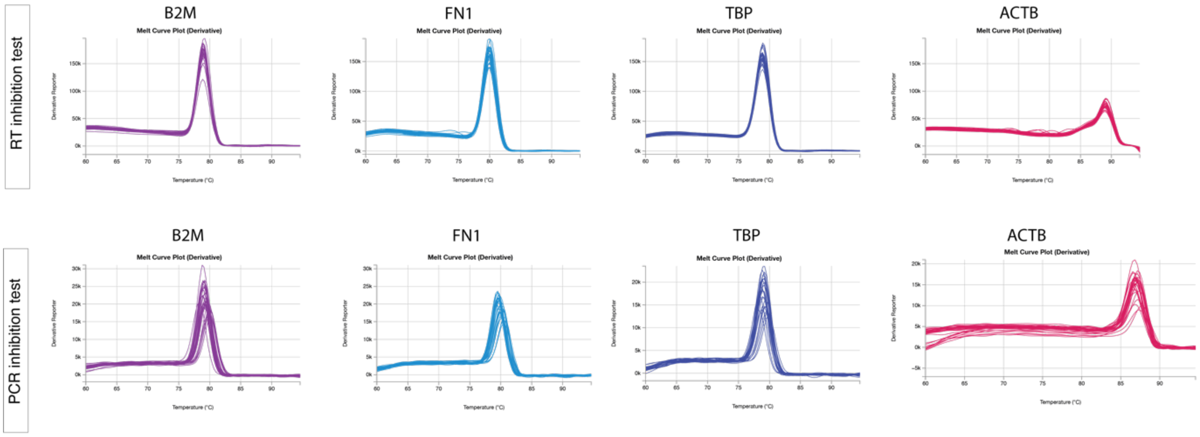

Figure S8. The specificity of RT and PCR in the presence of dextran and Xanthan gum biopolymers. Each graph is represented by multiple melting curves superimposed on each other and corresponds to different biopolymer concentration and type (dextran and XG), as indicated in Figure 4. For the RT inhibition test (top row) Maxima H minus RT enzyme was used followed by qPCR (Maxima Hot Start Taq DNA Polymerase). For the PCR inhibition test (bottom row) Maxima Hot Start Taq DNA Polymerase enzyme was used. The presence of biopolymer had minimal or no impact on the melting temperature of the amplicon thereby indicating high specificity of DNA amplification. The qPCR targets were B2M, FN1, TBP, and ACTB. Further details can be found in the Material and Methods section.
